# Congenital Supravalvar Mitral Ring - A Case Report

**DOI:** 10.21470/1678-9741-2018-0403

**Published:** 2020

**Authors:** Raquel Reis Soares, Leonardo Ferber, Matheus Ferber, Daniel Soares Mata

**Affiliations:** 1 Department of Anesthesiology, Biocor Instituto, Nova Lima, MG, Brazil.; 2 Department of Cardiovascular Surgery, Hospital das Clínicas da Universidade Federal de Minas Gerais, Belo Horizonte, MG, Brazil.; 3 Faculdade de Medicina de Barbacena, Barbacena, MG, Brazil.

**Keywords:** Mitral Valve Stenosis. Heart Defects, Congenital. Systole. Arteries. Mitral Valve Stenosis. Blood Pressure. Prognosis

## Abstract

Congenital mitral valve stenosis is a rare and severe disease, usually associated with other heart defects. The appropriate intervention depends on the site and mechanism of valvular obstruction and the aim is to avoid or delay valve replacement since it is associated with significant morbidity and mortality. Early single-stage complete repair is associated with better prognosis. We report the case of a 20-month-old child with a supravalvar mitral ring combined with a ventricular septal defect; pulmonary arterial systolic pressure before the surgery was 79 mmHg. The patient underwent a successful surgical repair with good clinical resolution.

**Table:** 

Abbreviations, acronyms & symbols	
CHD	= Congenital heart disease
CMS	= Congenital mitral valve stenosis
LA	= Left atrium
LAP	= Left atrial pressure
LV	= Left ventricle
LVP	= Left ventricular pressure
NO	= Nitric oxide
PaCO_2_	= Arterial partial pressure of carbon dioxide
PH	= Pulmonary hypertension
RV	= Right ventricle
SVMR	= Supravalvar mitral ring

## INTRODUCTION

Mitral ring is a subtype of congenital mitral valve stenosis (CMS) and is part of a spectrum of obstructive lesions affecting the left heart^[1]^. Rarely isolated^[2]^, mitral ring is usually associated with various congenital heart defects^[3]^, such as interventricular communication, coarctation of the aorta, subaortic stenosis, parachute mitral valve, left superior vena cava draining in the tectum of the left atrium, and *cor triatriatum* ^[4]^. The variability of the pathology and the defects interfere with the best strategy choice. Supramitral ring is usually associated with a normal valve apparatus^[5]^. Special emphasis is placed on the differential diagnosis, since early recognition of the lesion may enable the patient to benefit from surgical intervention^[6]^.

## CASE REPORT

**Fig. 1:**
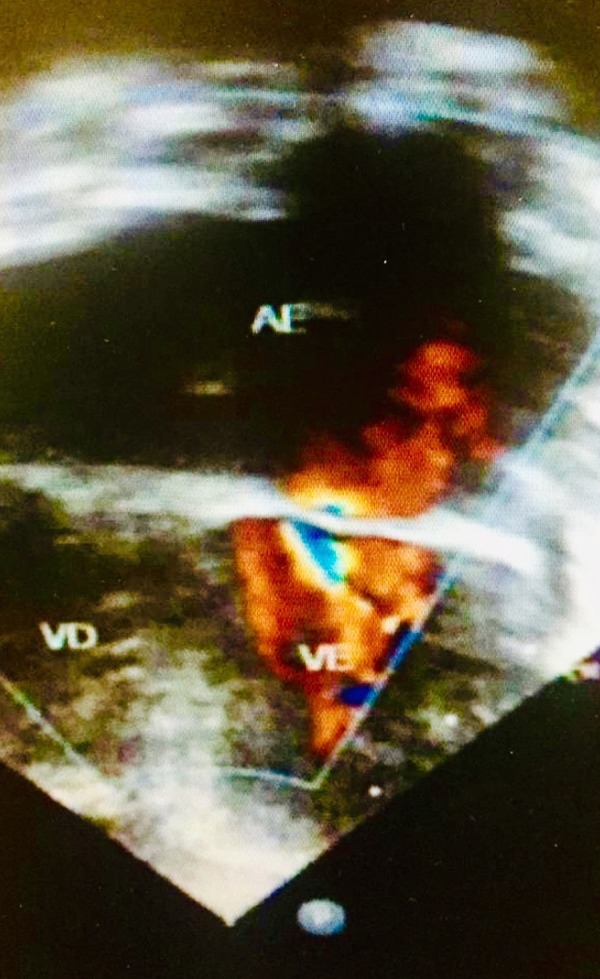
Two-dimensional echocardiography showing supravalvar mitral ring 225 x 400mm. AE=left atrium; VE=left ventricle; VD=right ventricle

A 20-month-old child was referred to our institution with a diagnosis of CMS. He experimented progressive dyspnea on efforts and nocturnal dyspnea. His weight was consistently below the 5^th^ percentile. Transthoracic echo evaluation showed a supravalvar mitral ring (SVMR) associated with a large inlet ventricular septal defect (Figure 1). In order to evaluate cardiac pressures and to test pulmonary reactivity to inhaled nitric oxide (NO), the patient was submitted to a cardiac catheterization. The systolic pulmonary artery pressure was 79 mmHg and the mean mitral valve inflow pressure gradient was 25 mmHg. Careful administration of inhaled NO for five minutes was done to test pulmonary reactivity. After the administration of 20 parts per million of inhaled NO, the systolic pulmonary artery pressure was significantly decreased (42 mmHg).

**Fig. 2:**
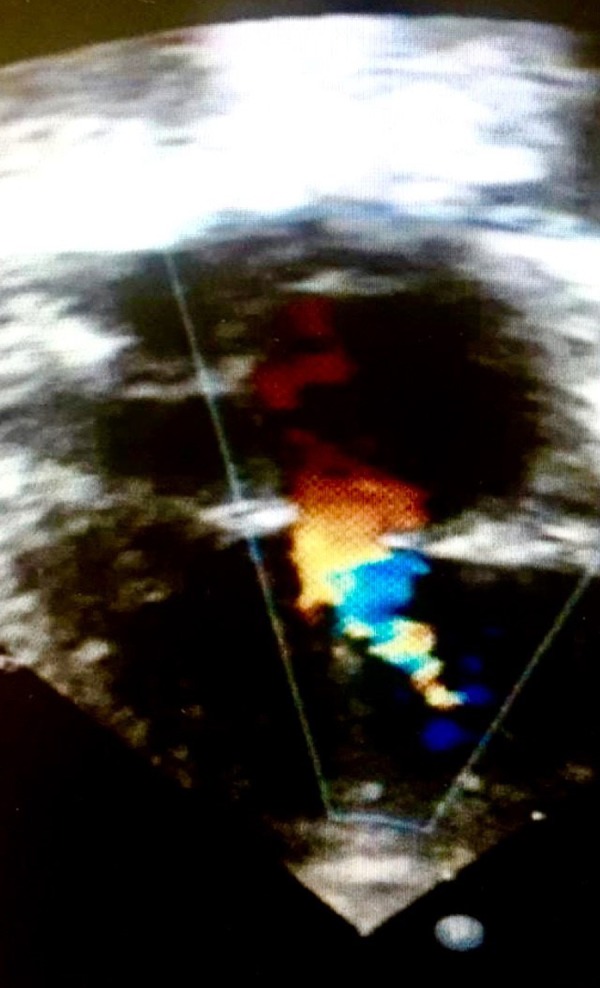
Two-dimensional echocardiography showing a postoperative image of surgical repair of supravalvar mitral ring stenosis. 169 x 300mm.

The patient was submitted to a surgical resection of the SVMR with closure of the ventricular septal defect (Figure 2). The mitral valve was normal. In order to reduce the pulmonary arterial hypertension, he received milrinone and a careful ventilation, to avoid hypercarbia and hypoxemia. The postoperative pulmonary hypertension (PH) was treated with sedation for 72 hours, NO administered as an inhaled gas, increased inspired oxygen fraction, moderated hyperventilation (arterial partial pressure of carbon dioxide [PaCO_2_] between 30-35 mmHg), and moderate alkalosis (pH>7.5). The final left atrial pressure to left ventricular pressure (LAP-LVP) gradient was 3 mmHg and the final systolic pulmonary artery pressure was 30 mmHg.

He was discharged from the hospital with resolution of both mitral stenosis and ventricular septal defect and a lower pulmonary artery pressure, treated ambulatorially with oral sildenafil.

## DISCUSSION

Various malformations result in CMS, associated or not with other cardiac defects. It affects 0,4% of patients with congenital heart disease (CHD)^[7]^. Anatomic treatments for CMS include balloon mitral valvuloplasty, surgical mitral valvuloplasty, and mitral valve replacement. A detailed assessment of the morphology and hemodynamic alteration will define the best treatment strategy. Early recognition of the lesion may anticipate the surgical intervention^[6]^.

SVMR is rarely seen in neonates, the ring develops and often progress during infancy^[8]^. It is an abnormal ridge of connective tissue arising from the left atrial wall and ranges from a thin membrane to a thick discrete ridge^[5]^. It partially obstructs the mitral valve inflow and increases venous capillary pressure and pulmonary artery pressure due to an elevated LAP. Congestive heart failure is expected to occur, as it can be seen in this case. Intervention is indicated in the most severe cases to improve mitral valve function^[9]^. Among those in which the mitral apparatus is normal, the outcome is better^[8]^.

Surgical repair is the preferred treatment for SVMR stenosis, mainly in association with other cardiac lesions. The prognosis in those who require resection within the first 18 months of life is poor due to high mortality and recurrent supravalvar mitral stenosis^[1]^. There is a trend toward early single-stage complete repair^[10]^. Mitral valve replacement and young age are associated with worse survival^[7,10-12]^.

In this case, SVMR was associated with ventricular septal defect and pulmonary arterial hypertension. Besides surgical repair, pulmonary arterial hypertension control was the key to success. The distinction between pulmonary vasoconstriction and pulmonary vascular occlusive disease will influence the prediction as to whether a child with PH in association with CHD will respond to the management strategies^[13]^. The pulmonary vasoreactivity test should be done to define the best candidates for heart surgery among patients with CHD and pulmonary arterial hypertension^[14]^.

Endothelial injury associated with cardiopulmonary bypass in patients with CHD predisposes to development of PH. In this patient, left to right shunt and anatomic factors that impose obstruction to pulmonary blood flow (SVMR) were present. After surgical repair, attempts to reduce pulmonary vascular resistance were employed: increased inspire oxygen fraction, moderate hyperventilation, and moderate alkalosis. Pharmacologic interventions were: milrinone, a phosphodiesterase inhibitor, and NO, an endothelium-derived vasodilator with no systemic effect when inhaled^[15]^. In order to avoid rebound PH, NO was careful reduced till the patient was extubated^[16]^.

Mitral valve repair for congenital supravalvar ring stenosis can be performed successfully. This delicate population of patients should be evaluated looking for other heart defects. Ring resection leads to excellent long-term result^[8]^. Special attention must be paid to children in risk of PH. Good early survival depends on single-stage repair and postoperative management strategies for PH.

**Table:** 

Author's roles & responsibilities	
RRS	Substantial contributions to the conception or design of the work; or the acquisition, analysis, or interpretation of data for the work; final approval of the version to be published
LF	Substantial contributions to the conception or design of the work; or the acquisition, analysis, or interpretation of data for the work; final approval of the version to be published
MF	Substantial contributions to the conception or design of the work; or the acquisition, analysis, or interpretation of data for the work; final approval of the version to be published
DSM	Substantial contributions to the conception or design of the work; or the acquisition, analysis, or interpretation of data for the work; final approval of the version to be published
